# Functional characterization of electron-transferring flavoprotein and its dehydrogenase required for fungal development and plant infection by the rice blast fungus

**DOI:** 10.1038/srep24911

**Published:** 2016-04-26

**Authors:** Ya Li, Jindong Zhu, Jiexiong Hu, Xiuli Meng, Qi Zhang, Kunpeng Zhu, Xiaomin Chen, Xuehang Chen, Guangpu Li, Zonghua Wang, Guodong Lu

**Affiliations:** 1 Key Laboratory of Biopesticides and Chemical Biology, Ministry of Education, Fujian Agriculture and Forestry University, Fuzhou, Fujian, 350002, China; 2 Department of Biochemistry and Molecular Biology, University of Oklahoma Health Sciences Center, Oklahoma City, OK 73104, USA

## Abstract

Electron-transferring flavoprotein (ETF) and its dehydrogenase (ETFDH) are highly conserved electron carriers which mainly function in mitochondrial fatty acid β oxidation. Here, we report the identification and characterization of ETF α and β subunit encoding genes (*ETFA* and *ETFB*) and ETFDH encoding gene (*ETFDH*) in the rice blast fungus *Magnaporthe oryzae*. It was demonstrated that, by impacting fatty acid metabolism, ETF and ETFDH mutations led to severe growth and conidiation defects, which could be largely rescued by exogenous acetate or carbonate. Furthermore, although conidium germination and appressorium formation appeared to be normal in ETF and ETFDH mutants, most appressoria failed to penetrate the host epidermis due to low turgor pressure. The few appressoria that succeeded in penetration were severely restricted in invasive growth and consequently failed to cause disease. Moreover, ETF mutant *etfb*^*−*^ induced ROS accumulation in infected host cells and exogenous antioxidant GSH accelerated mutant invading growth without increasing the penetration rate. In addition, mutant *etfb*^*−*^ displayed elevated lipid body accumulation and reduced ATP synthesis. Taken together, ETF and ETFDH play an important role in fungal development and plant infection in *M. oryzae* by regulation of fatty acid metabolism, turgor establishment and induction of host ROS accumulation.

Rice blast caused by *Magnaporthe oryzea* (*M. oryzae*) is a destructive disease in rice-planting areas worldwide, and poses a severe threat to global food security, especially in the current changing climate^1^,^2^. *M. oryzae* has emerged as a model organism for the study of plant fungal pathogens and their interaction with hosts^3^,^4^,^5^. *M. oryzae* infection starts with a three-celled conidium contacting the host surface, which subsequently germinates and develops into a specialized structure called the appressorium^6^. The appressorium matures by melanin-pigmentation and produces enormous turgor pressure by accumulating high concentrations of glycerol^7^,^8^. The high pressure promotes the formation of a narrow penetration peg that elongates from the appressorium base and mechanically penetrates the host cuticle^9^. Next, invasive hyphae (IH) develop from the penetration peg and propagate from one cell to another via plasmodesmata in susceptible hosts^10^. After colonization for 4–5 days, characteristic blast lesions appear on the host surface, where conidia are produced and spread to start a new infection cycle^11^.

The initial infection processes at the host surface from conidia adhesion to appressorial development do not require nutrient acquisition by the fungus, but does require the mobilization and metabolism of the storage reserves in conidia^12^. During this stage, lipid droplets move into incipient appressorium and coalesce into a central vacuole at the onset of turgor generation, which is under the control of the Pmk1 MAP kinase pathway^13^. Along this line, rapid lipolysis catalyzed by triacylglycerol lipases release large amounts of glycerol in a process regulated by the cAMP-dependent protein kinase A^13^. The glycerol concentration can reach a very high level over 3.0 M, which is believed to directly generate hydrostatic pressure by drawing water into the appressorium cell^14^. Simultaneously, the melanin layer is formed in the outer appressorium to prevent glycerol efflux and maintain the high turgor pressure^7^.

In addition to glycerol, lipolysis also generates fatty acids that enter the β oxidation pathway for breakdown in either mitochondria or peroxisomes depending on the carbon chain length of the fatty acids^15^,^16^. The short and medium-chain fatty acids with less than 20 carbons are mainly oxidized in mitochondria^17^, while long-chain fatty acids of over 20 carbons are first degraded into short-chain fatty acids in peroxisomes, which then move into mitochondria for complete oxidation^18^. The first dehydrogenation step of mitochondrial fatty acid β oxidation is catalyzed by acyl-CoA dehydrogenases. Besides catalyzing dehydrogenation, acyl-CoA dehydrogenases also transfer electrons to an electron-transferring flavoprotein (ETF), which, through an electron-transferring flavoprotein dehydrogenase (ETFDH), finally delivers the electrons to the ubiquinone pool in the terminal respiratory system for ATP synthesis^19^,^20^. Thus, ETF and ETFDH link the fatty acids oxidation with respiratory system. To date, functional studies of ETF and ETFDH are well reported in humans, animals and yeasts. ETF consists of α- and β- subunits which contain a FAD cofactor and an AMP molecule respectively, while ETFDH is a monomer containing a FAD molecule and an iron–sulfur cluster^21^,^22^. Inherited mutations of ETF and/or ETFDH can result in multiple acyl-CoA dehydrogenase deficiency (MADD) and disrupt fatty acid β oxidation leading to a series of metabolic disorders^19^,^23^. Yeast ETF and ETFDH homologs Aim45, Cir1 and Cir2 were involved in cellular redox state under stress conditions^24^.

The roles of ETF and ETFDH are not well understood in filamentous fungi. In the current study, we first identify and functionally characterize two ETF genes (*ETFA* and *ETFB*) and one *ETFDH* gene in the rice blast fungus *M. oryzae* by targeted gene deletion and/or T-DNA insertion mutation. Our data demonstrate that both ETF and ETFDH play an important role in vegetative growth, conidiation and infection-related development of *M. oryzae* through regulation of fatty acid metabolism, turgor establishment and host ROS accumulation.

## Results

### Identification of ETF and ETFDH in *M. oryzae*

**Figure 1:**
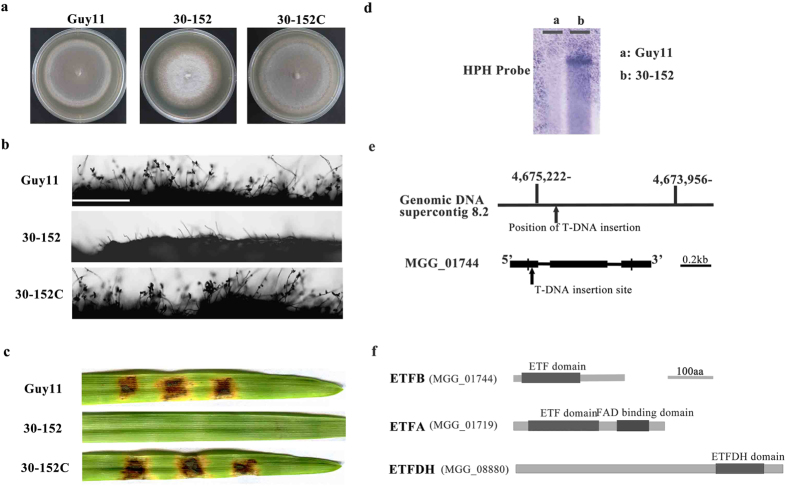
Identification of ETFB by T-DNA insertion mutation and domain structure of ETFA, ETFB and ETFDH in *M. oryzae*. (**a**) Colony morphology of insertion mutant 30-152 grown on CM medium for 12 days. The 30-152 colony has reduced pigmentation relative to Guy11 and complemented strain (30-152C). (**b**) Microscopic observation of mutant 30-152 sporulation. 30-152 failed to produce conidia when grown on CM. Bar, 100 μm. (**c**) Pathogenicity assay of mutant 30-152 on barley by inoculated with mycelial blocks. No disease lesion was caused by the mutant after inoculation for 5 days. (**d**) Southern blot detecting the T-DNA insertion copy in mutant 30-152. Only one band was detected by the *HPH* probe in the mutant genome digested with *EcoR* І. (**e**) T-DNA insertion site identification in the mutant 30-152. The coding region of *ETFB* (MGG_01744) was disrupted by T-DNA 123 bp downstream of the translation initiation site. (**f**) The domain structure of *M. oryzae* ETFA, ETFB and ETFDH as annotated at the Broad Institute of MIT (http://www.broadinstitute.org/).

To identify functional genes in *M. oryzae*, a T-DNA insertion-mutation library containing over 1000 hygromycin-resistant transformants was constructed by *Agrobacterium tumefaciens*-mediated transformation (ATMT) as described previously^25^. Phenotype analysis revealed a pale mutant (30-152) that failed to produce conidia on complete medium CM in comparison to the wild type strain Guy11 (Fig. 1a,b). Furthermore, the mutant mycelia did not cause disease symptoms on barley leaves (Fig. 1c). Southern blot results indicated that only one T-DNA copy was integrated in the 30-152 genome (Fig. 1d), suggesting a single gene disruption. Hi-Tail PCR was carried out and subsequent sequencing analysis indicated that the T-DNA insertion site in 30-152 was located 123bp downstream of the translation initiation site of the ETF β subunit encoding gene *ETFB* (MGG_01744.8, Broad Ins.) on supercontig 8 of chromosome II (Fig. 1e). The phenotype defects of 30-152 were fully restored when the full length of *ETFB* gene was reintroduced into 30-152 under the control of a 1.5Kb native promoter sequence (Fig. 1a–c).

Based on sequence homology to *ETFB*, we further identified an *ETFA* gene (MGG_01719.8, encoding ETF α subunit) and an *ETFDH* gene (MGG_08880.8, encoding ETF dehydrogenase) in the *M. oryzae* genome (Fig. 1f). ETFB and ETFDH contained an ETF domain and an ETFDH domain, respectively (Fig. 1f), while ETFA also contained a FAD binding domain in addition to the ETF domain (Fig. 1f). Phylogenetic analysis of ETF and ETFDH sequences from several model fungi, bacteria, plants and animals indicated that the amino acid sequences of ETFA, ETFB and ETFDH of *M. oryzae* are highly conserved among filamentous fungi and animals by displaying over 50% similarity, despite a slow decrease in yeast, bacteria and plants (Fig. S1). However, the functions of ETF and ETFDH in filamentous fungi have not been well characterized.

### ETF and ETFDH mutants are defective in vegetative growth and conidiation

To investigate the functions of the two ETF genes (*ETFA* and *ETFB*) and the ETFDH gene (*ETFDH*) in *M. oryzae*, we conducted targeted gene deletion mutagenesis and obtained at least two mutants for each gene. The gene deletions were confirmed by Southern blot analysis (Fig. S2). Because the two mutants for each gene were the same phenotypically, only one was selected for further characterization and named *etfa*^*−*^, *etfb*^*−*^ and *etfdh*^*−*^ respectively. In addition, over 10 complementation transformants were generated for each mutant and were able to rescue the phenotype defects of each mutant, they were named *etfa*^*−*^*/ETFA*, *etfb*^*−*^*/ETFB*, and *etfdh*^*−*^*/ETFDH* respectively.

**Figure 2:**
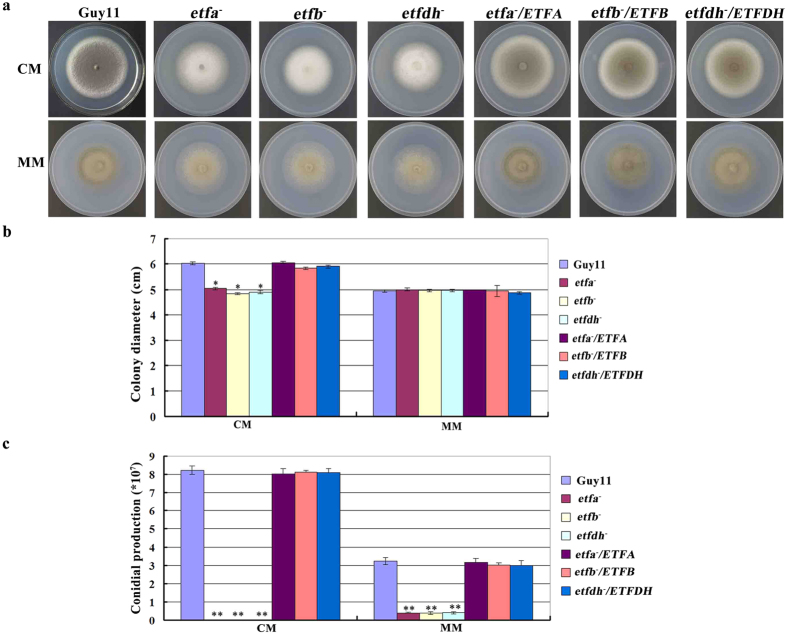
ETF and ETFDH mutants displayed growth and conidiation defects. (**a**) The colonial morphology of ETF and ETFDH mutants grown on different medium for 10 days. Colonies of ETF and ETFDH mutants lack pigmentation and grew more slowly on CM than on MM, RBM and SDC. CM, complete medium; MM, minimal medium; RBM, rice bran medium; SDC, straw decoction-corn medium. (**b**) Bar chart showing the growth rate of ETF and ETFDH mutants. The growth rate of mutants had no significant change from wild type on MM, RBM and SDC medium. Mean and standard deviation were calculated from three independent replicates. Significant differences are indicated by asterisks (*P < 0.05; t test). (**c**) Bar chart showing conidial production of ETF and ETFDH mutants cultured on different media. All strains grew for 12 days and conidia were harvested into 1 ml of ddH_2_O for counting. Mutants completely failed to produce conidia on CM and formed about 10% of the amount of conidia of Guy11 on MM, RBM and SDC media. Mean and standard deviation were calculated from three independent replicates. Significant differences are indicated by double asterisks (**P < 0.01; t test).

Because of the roles of ETF and ETFDH in fatty acid β oxidation and nutrition metabolism, we assessed the growth and conidiation of the deletion mutants by growing on two different nutrition media, complete medium (CM) and minimal medium (MM). On the CM, the colonies of ETF and ETFDH mutants (*etfa*^*−*^, *etfb*^*−*^ and *etfdh*^*−*^) completely lacked pigmentation in comparison to the grey colonies of Guy11 and the complemention strains (Fig. 2a). In addition, the colony size of ETF and ETFDH mutants was reduced (from ~6.0cm to ~5.0cm in diameter) after a 10-day growth period (Fig. 2b). These results were similar to those of the insertion mutant 30-152 on CM. However, when cultured on MM, ETF and ETFDH mutants only displayed a slight pigmentation reduction and the mutant colony size was similar to Guy11 (Fig. 2a,b). Growth assays on rice bran medium (RBM) and straw decoction-corn (SDC) medium yielded the same results as on MM (Fig. S3a,b).

Furthermore, no conidia were produced by ETF and ETFDH mutants growing on CM, in contrast to the numerous conidia (6–8 × 10^7^/plate) produced by Guy11 and the complemention strains after incubation for 12 days (Fig. 2c). However, when growing on MM, RBM or SDC medium, ETF and ETFDH mutants produced about 8–10% conidia of Guy11 (Figs 2c and S3c). We speculated the difference between CM and other simple media of MM, RBM and SDC likely induced different nutrition stress on the mutants. These results suggested that ETF and ETFDH were involved in growth and conidiation by *M. oryzae*.

### ETF and ETFDH mutants are defective in fatty acid metabolism

**Figure 3:**
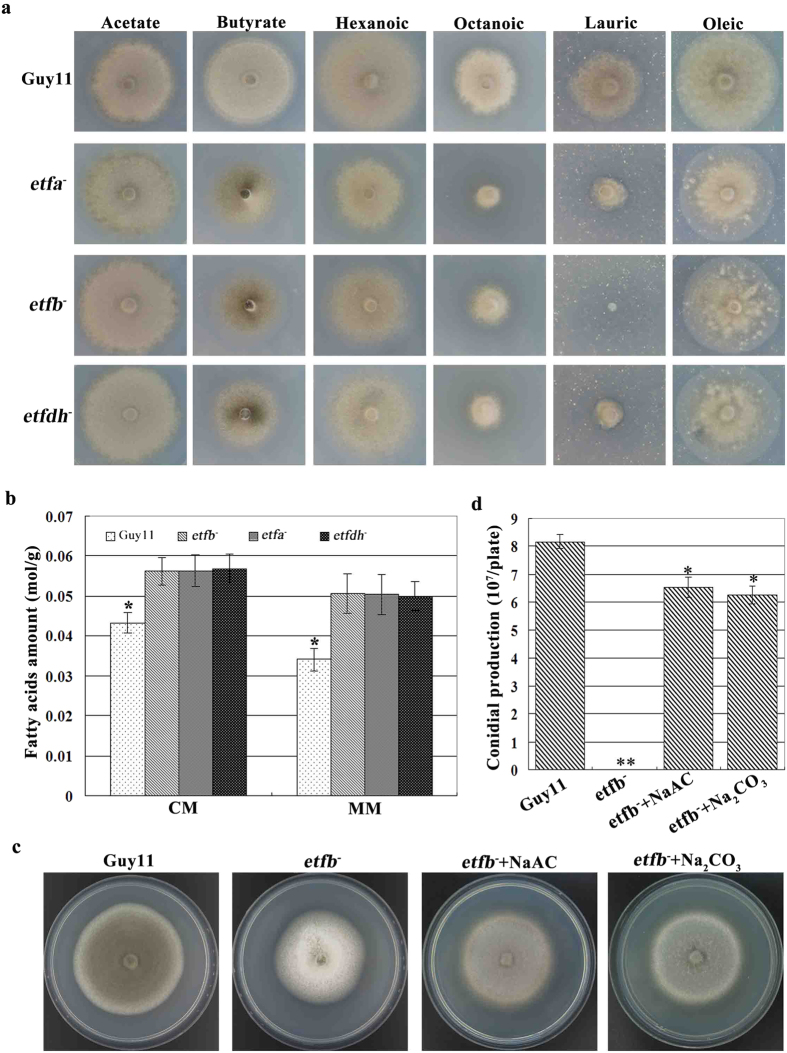
ETF and ETFDH were involved in fatty acids metabolism. (**a**) The growth of ETF and ETFDH mutants on MM with various fatty acids as sole carbon source after incubation for 10 days. Mutant growth was significantly reduced on butyrate (C4, 10 mM), hexanoic (C6, 2.5 mM), octanoic (C8, 1 mM) or lauric (C12, 2.5 mM) as sole carbon source. The growth of mutants was not affected on acetate (C2, 50 mM) and oleic (C18, 2.5mM) as sole carbon source. (**b**) Bar chart showing the total fatty acids amount of ETF and ETFDH mutants by growing on CM and MM. Mutants generated more fatty acids on CM than MM. Mean and standard deviation were calculated from three independent replicates. Significant differences are indicated by asterisks (*P < 0.05; t test). (**c**) Colony pigmentation and growth defects of mutant *etfb*^*−*^ were largely suppressed by adding NaAC and Na_2_CO_3_ to CM medium for 10 days. (**d**) The conidial production of mutant *etfb*^*−*^ was greatly rescued by adding NaAC (50 mM) and Na_2_CO_3_ (50 mM) to CM medium and incubated for 12 days. Mean and deviation were calculated from three independent replicates. Significant differences are indicated by asterisks (*P < 0.05; **P < 0.01; t test).

As reported in human, ETF and ETFDH mutation could impact upstream fatty acid metabolism and cause metabolic diseases^19^,^23^. To investigate the effect of *M. oryzae* ETF and ETFDH deletion on fatty acid metabolism, we determined the mutant ability to utilize exogenous fatty acids as the sole carbon source. Since mitochondrial β oxidation of fatty acids is generally responsible for degrading fatty acids with <20 carbons^17^, several fatty acids with different carbon length from C2 to C18 were added to the carbon-deficient MM medium to test the fungal growth. Our results showed that, when growing on butyrate (C4), hexanoic (C6), octanoic (C8) or lauric (C12) as sole carbon source, the growth of ETF and ETFDH mutants was apparently inhibited in comparison to that of Guy11 (Fig. 3a). However, when growing on acetate (C2) and oleic (C18) as sole carbon source, ETF and ETFDH mutants displayed no growth difference from Guy11 (Fig. 3a).

To further investigate the change of endogenous fatty acid metabolism, we determined the total amounts of fatty acids in ETF and ETFDH mutants growing on CM and MM. Our results showed that the mutants consistently accumulated more fatty acids than Guy11 on both CM and MM (Fig. 3b). Furthermore, ETF and ETFDH mutants produced more fatty acids on CM than on MM (Fig. 3b), which may explain our observation that the mutants showed more severe growth and conidiation defects on CM.

To further understand the impact of fatty acids accumulation on the growth and conidiation of *M. oryzae*, we treated ETF and ETFDH mutants with sodium acetate (NaAc) to rescue the mutant defects. Our results showed that this treatment almost fully recovered the colony color and growth rate of the mutants (Figs 3c and S4a). Moreover, the conidial production of ETF and ETFDH mutants was largely restored when growing on NaAc-treated CM (Figs 3d and S4b). In addition, the carbonate Na_2_CO_3_ displayed similar effect to NaAc in rescuing the mutant defects (Figs 3c,d and S4).

### ETF and ETFDH mutants are defective in pathogenicity

**Figure 4:**
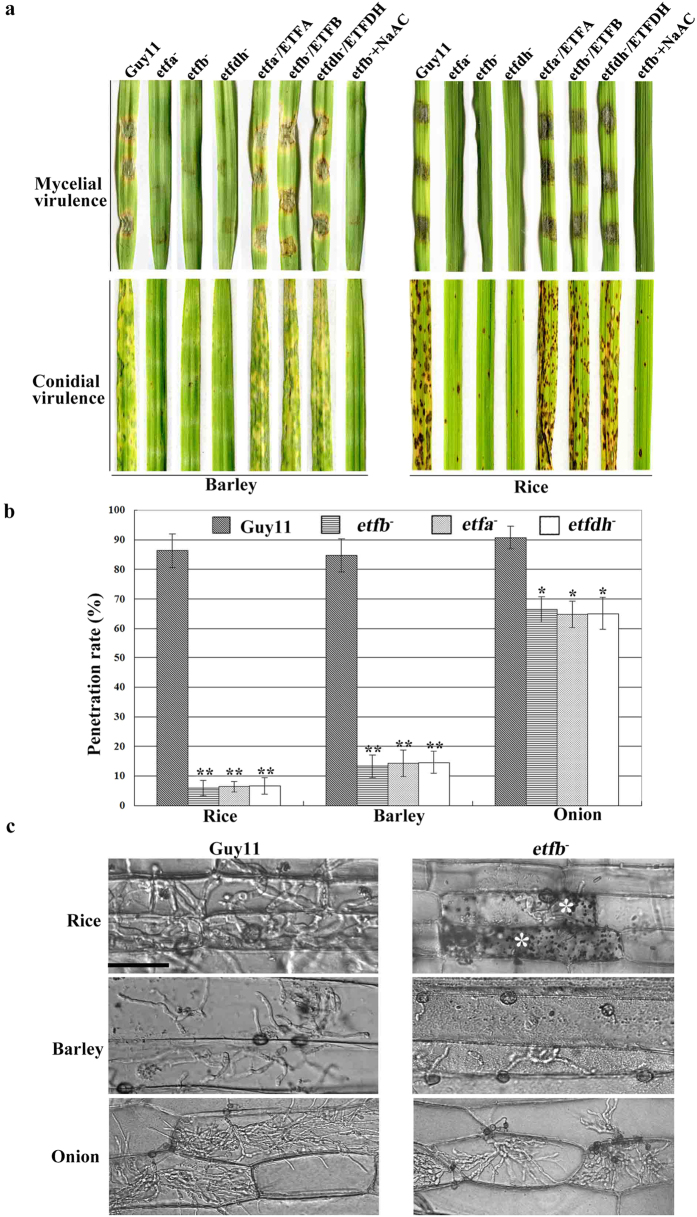
ETF and ETFDH mutants were defective in pathogenicity. (**a**) Mycelial virulence was tested by inoculating mycelial blocks from CM culture on excised rice and barley leaves for 6 days. Mycelia of ETF and ETFDH mutants failed to infect barley and rice. Conidial virulence was tested by harvesting conidia from SDC culture at a concentration of 5 × 10^4^ spores/ml and spraying on live barley and rice seedlings before incubation for 6 days. Mutant conidia were also unable to infect live barley and rice. Exogenously adding NaAC could not recover the virulence of mycelia or conidia of the *etfb*^*−*^ mutant. (**b**) Bar chart showing penetration rate on rice sheath, barley leave epidermis and onion bulb epidermis after incubation for 48 h using conidia. ETF and ETFDH mutants displayed differential penetration rate on different host surface. Mean and deviation were calculated from three independent replicates. Over 100 appressoria was counted for calculating the penetration rate in one replicate. Significant differences are indicated by stars (*P < 0.05; **P < 0.01; t test). (**c**) The growth of *etfb*^*−*^ invasive hyphae (IH) on rice sheath, barley leaf epidermis and onion bulb epidermis after incubation for 48 h. Mutant IH growth was inhibited in to different degrees depending on the host. In the rice sheath assay, the mutant IH was surrounded by microbodies in infected cells (indicated by asterisks, *). Bar, 50 μm.

Mitochondrial β oxidation has been reported to be involved in infection-related development in plant pathogenic fungi^26^, but the role of downstream electron transfer flavoprotein in pathogenicity is still unknown. So we set to clarify whether the ETF and ETFDH mutations could affect the pathogenicity of *M. oryzae*. As ETF and ETFDH mutants did not produce conidia on CM, we used mycelia blocks to inoculate excised barley and rice leaves. Our results showed that the mutant mycelia completely failed to cause disease on barley and rice (Fig. 4a), consistent with the result of the insertion mutant 30-152. In this regard, NaAc was unable to recover the pathogenicity of the mutants.

As a small number of conidia were formed by ETF and ETFDH mutants growing on MM, RBM and SDC medium, we tested the conidial germination and appressoria formation on hydrophobic surface but did not find any variations (Fig. S5a). However, by spraying onto live barley and rice seedlings, the mutant conidia were almost completely non-pathogenic, despite producing a few non-extended necroses on the host surface; in comparison to the typical blast lesions produced by Guy11 and the complemention strains (Fig. 4a). Again, NaAc treatment was unable to recover the pathogenicity of the mutant conidia (Fig. 4a).

We further examined infection sites on rice sheath and barley epidermis and found that about 6% of the mutant appressoria could penetrate rice sheath cells (Fig. 4b) and the growth of invasive hyphae (IH) was severely impaired relative to Guy11 (Figs 4c and S5b). The mutant IH was surrounded by microbodies in the rice cell (Figs 4c and S5b). On barley epidermis, about 14% mutant conidia could complete penetration (Fig. 4b), but the mutant IH still displayed limited extension (Figs 4c and S5b). Onion epidermis was also used to detect the mutant penetration and extension in plant cells. Interestingly, the mutant displayed a much higher penetration rate of over 65%, although the IH extension was still more limited than for Guy11 infection (Figs 4b,c and S5b). These results suggested that the pathogenicity defects of ETF and ETFDH mutants may result from inefficient penetration, restricted IH extension and induction of greater defense responses in rice compared to onion.

### ETF mutant *etfb* ^*−*^ exhibits reduced turgor pressure

**Figure 5:**
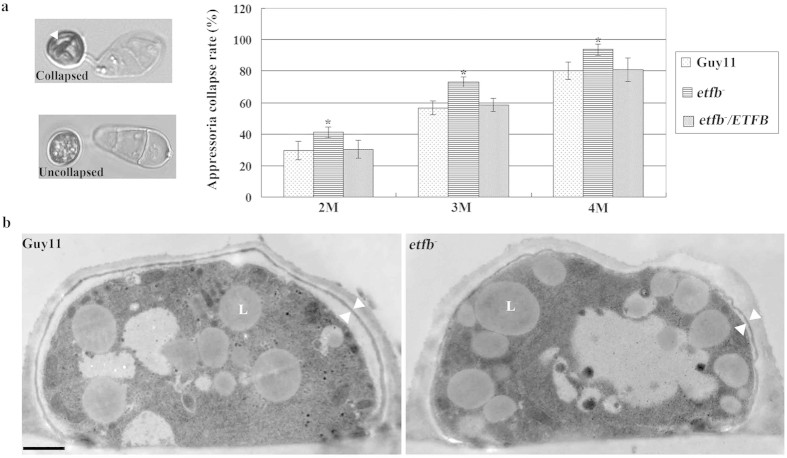
ETF mutant *etfb*^*−*^ was impaired in appressorium turgor and melanin layer. (**a**) Bar chart showing the frequency of appressorium collapse of ETF mutant *etfb*^*−*^ by treating matured appressoria with 2M, 3M and 4M glycerol. Mutant *etfb*^*−*^ displayed an elevated frequency of collapse at each glycerol concentration, indicating a lower turgor pressure compared to the wild type. Mean and standard deviation were calculated from three independent replicates. Over 100 appressoria was counted for computing collapse rate in one replicate. White arrows point to a collapsed appressorium in contrast to the uncollapsed example below. Significant differences are indicated by asterisks (*P < 0.05; t test). (**b**) Appressorium melanin layer observation of ETF mutant *etfb*^*−*^ by TEM. The appressorium was induced on rice sheath and then prepared for TEM. White arrows point to the melanin layer of the appressorium. ‘L’ indicates lipid body. Bar, 500 nm.

The enormous turgor pressure of the appressorium is essential for *M. oryzae* to penetrate the host epidermis and cause disease^27^. To determine whether the pathogenicity loss in mutant *etfb*^*−*^ resulted from turgor pressure defect, we conducted the cytorrhysis assay by treating appressoria with 2 M, 3 M and 4 M glycerol and quantifying the collapsed cells. Our results showed that mutant *etfb*^*−*^ always displayed a higher collapsed rate than Guy11 at each glycerol concentration (Fig. 5a), suggesting lower turgor pressure in mutant appressoria. As the plant hosts may be different in surface properties or hardiness, the reduced turgor pressure in mutant may well account for the different penetration rate on rice, barley and onion.

In addition to glycerol accumulation, lipid body mobilization from conidia to appressorium is also necessary for building up turgor pressure^13^. To this end, we determined whether the upstream lipid body mobilization was altered in mutant *etfb*^*−*^ by staining with the lipophilic fluorescent probe Bodipy and monitoring the movement and localization of Bodipy-labeled lipid bodies by fluorescence microscopy. Our results showed that there was no difference between mutant *etfb*^*−*^ and Guy11 in the mobilization and movement of lipid bodies into the appressorium (Fig. S6), suggesting that the appressorial turgor defect in mutant was not due to alterations in lipid mobilization.

To further understand the mechanism of turgor reduction in the mutants, we examined the appressorial melanin layer which is necessary for turgor generation by preventing glycerol from leaking out^7^. Appressoria were induced on rice sheaths for 24 h and processed for transmission electron microscopy. As shown in Fig. 5b, the melanin layer of mutant *etfb*^*−*^ appressorium was obviously thinner than that of Guy11 strain. Therefore, we concluded that the low turgor pressure in mutant likely resulted from an impaired appressorial melanin layer.

### ETF mutant *etfb* ^*−*^ is more sensitive to host oxidative stress

**Figure 6:**
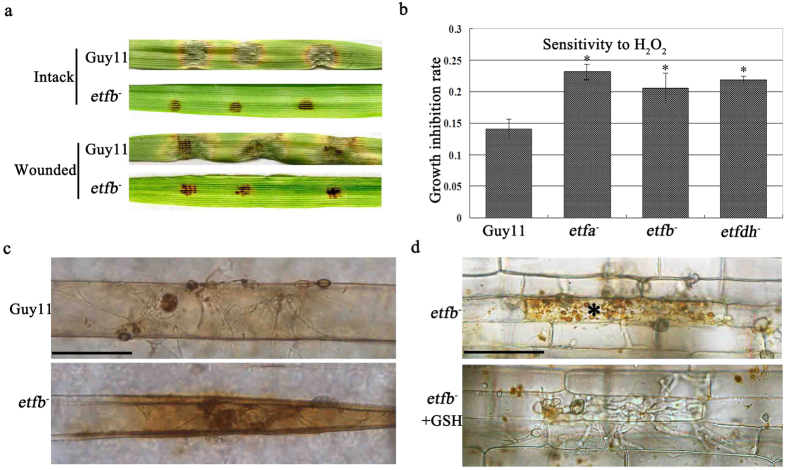
ETF mutants failed to overcome host oxidative stress. (**a**) Wounded barley leaves inoculated by mutant *etfb*^*−*^ with 20 μl conidial droplets. Infection by mutant *etfb*^*−*^ did not improve on a wounded host. The wound was made by gently scratching the barley leaves with a toothpick and then incubated for 6 days. (**b**) Bar chart showing the growth inhibition of ETF and ETFDH mutants by growing on CM medium with 0.5 mM H_2_O_2_. The growth inhibition rate was calculated by subtracting the colony diameter of H_2_O_2_ treatment from the normal colony diameter and dividing by normal colony diameter. Mutant growth was significantly inhibited by H_2_O_2_, indicating that the mutant was more sensitive to oxidative stress. Mean and standard deviation were calculated from three independent replicates. Significant differences are indicated by asterisks (*P < 0.05; t test). (**c**) DAB staining of infected barley leaves in mutant *etfb*^*−*^ after 24 h post-inoculation. The barley cell infected by the mutant displayed a more dark red color when stained by DAB (1 mg/ml), indicating a greater level of ROS accumulation. Bar, 50 μm. (**d**) Antioxidant GSH treatment of rice sheath infected by mutant *etfb*^*−*^. 5 mM GSH was added to rice sheath after inoculation for 24 h. The result was observed after additional incubation for 24 h. The microbodies surrounding mutant IH in infected cells were eliminated and the invasive growth of mutant was accelerated by GSH. Asterisks * indicate cells containing microbodies. Bar, 50 μm.

As described above, the reduced turgor pressure was responsible for the penetration defect in the mutants. However, a few of mutant appressoria could still penetrate but were limited in invasive hyphae growth. Moreover, mutant *etfb*^*−*^ remained non-pathogenic even on wounded host leaves (Fig. 6a), suggesting that the defects went beyond reduced turgor pressure and penetration. As reactive oxygen species induced by fungal infection of host plants could suppress fungal invasion and intracellular growth^28^,^29^, we determined the mutant ability to respond to host oxidative stress. We first tested the mutant *etfb*^*−*^ sensitivity to exogenous oxidative stress by adding 0.5mM H_2_O_2_ to the culturing CM and monitoring the growth rate, and found that the mutant growth was greatly reduced compared to Guy11 (Fig. 6b), which suggested an increased sensitivity to exogenous H_2_O_2_.

To further investigate how mutant *etfb*^*−*^ would respond to host-produced reactive oxygen species (ROS), we quantified the ROS accumulation in infected host cells using DAB staining. Our results showed that mutant *etfb*^*−*^ displayed a darker redish color at the infection site than Guy11 (Fig. 6c), which suggested excessive ROS accumulation. Moreover, when treated with glutathione GSH (an antioxidant against ROS), the microbodies surrounding the mutant IH were effectively cleared and the growth increased (Fig. 6d). However, GSH was unable to improve the mutant penetration rate (Fig. S7a). These results suggested that the invasive growth defect of the mutant was mainly due to the loss of ability to overcome the host oxidative stress.

### ETF and ETFDH proteins localize to mitochondria in *M. oryzae*

**Figure 7:**
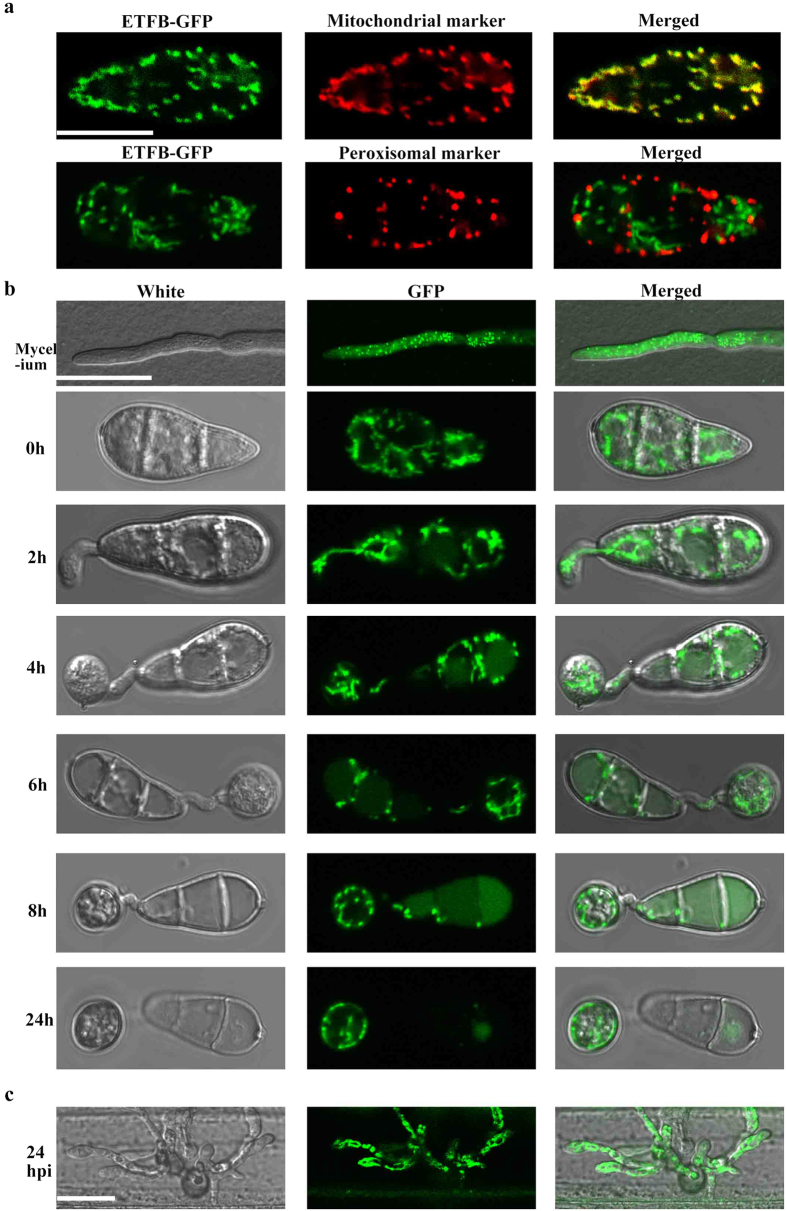
ETFB localized to mitochondria and expressed steadily in whole stage of *M. oryzae*. (**a**) Co-localization of ETFB-GFP to mitochondrial marker ATP-RFP and peroxisomal marker PTS1-RFP observed by confocal microscopy. ETFB-GFP almost completely co-localize with ATP-RFP, not PTS1-RFP, indicating a mitochondrial localization of ETFB. Bar, 10 μm. (**b**) ETFB-GFP expressed during mycelia growth, conidial germination and appressorium development. Bar, 10 μm. (**c**) The ETFB-GFP expression was detected during infection to barley epidermis at 24 hpi. Bar, 10 μm.

Fatty acid β oxidation occurs in both mitochondria and peroxisomes in most fungi^30^. ETF and ETFDH are predicted to exclusively function in the mitochondrial β oxidation pathway^19^, but there is no direct experimental evidence so far. Here, we determined the localization of *M. oryzae* ETF and ETFDH by co-localization with the mitochondrial marker ATP1-RFP and peroxysomal marker PTS1-RFP. Our results showed that ETFB-GFP almost completely co-localized with the mitochondrial marker ATP-RFP, but not with the peroxisomal marker PTS1-RFP at all (Fig. 7a). ETFA and ETFDH also localized to mitochondria (Fig. S8). These results suggested that ETF and ETFDH of *M. oryzae* specifically function in mitochondrial fatty acid β oxidation. Furthermore, we determined the spatio-temporal expression of ETFB by monitoring the GFP fluorescence intensity and found steady and high expression levels at all development stages including mycelial growth, conidiation, germination, appressorium formation (Fig. 7b) and even at the plant infection stage of 24 hpi (Fig. 7c).

### ETF mutant *etfb* ^*−*^ shows reduced ATP synthesis

**Figure 8:**
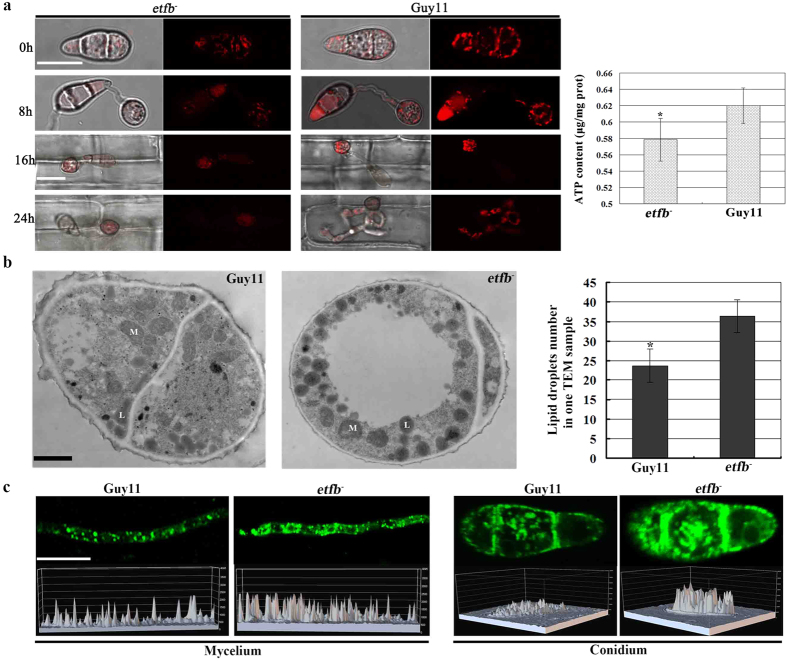
ETF mutant *etfb*^*−*^ showed reduced ATP synthesis and extensive lipid body accumulation. (**a**) The expression of ATP synthase in conidia, appressoria development and infection by detecting the ATP-RFP signal intensity. Mutant *etfb*^*−*^ displayed a weak ATP synthase expression observed by confocal microscopy. Bar, 10 μm. Bar chart showed the ATP content measured by HPLC. Mean and deviation were calculated from three independent replicates. Significant differences are indicated by asterisks (*P < 0.05; t test). (**b**) Observation of conidial lipid body in mutant *etfb*^*−*^ by TEM. More lipid droplets accumulated in conidial TEM sample of mutant. M indicates mitochondria; L indicates lipid body. Bar chart showed the average lipid body number in one TEM sample. Mean and deviation were calculated from three independent replicates. Over 20 separate TEM samples were counted in each replicate. Significant differences are indicated by asterisks (*P < 0.05; t test). Bar, 500 nm. (**c**) Observation of lipid body accumulation by staining with Bodipy. More lipid bodies were present and emitted brighter green fluorescence in mutant *etfb*^*−*^ mycelium and conidium detected by confocal microscopy. 3D chart showed the fluorescence distribution and intensity which was constructed by confocal analyzing software. High peak indicates high fluorescence intensity. Bar, 10 μm.

As electron carriers, ETF and ETFDH are required for downstream ATP synthesis catalyzed by ATP synthase^19^. Thus we determined the ATP synthase activity in the mutants by expressing a RFP tagged ATP synthase (ATP1) in *M. oryzae* as described previously^26^. By confocal fluorescence microscopy, we found that the ATP1-RFP expression signals in conidia, appressoria and infected cells were much lower in mutant *etfb*^*−*^ than in Guy11 (Fig. 8a). The results suggested lower ATP synthase activity in mutant *etfb*^*−*^. In addition, the intracellular ATP levels of mutant *etfb*^*−*^ were determined by HPLC. Our results showed that the ATP levels in mutant *etfb*^*−*^ were slightly reduced to ~0.58 μg/mg protein, in comparison to ~0.62 μg/mg protein in Guy11 (Fig. 8a). However, exogenously added ATP was unable to rescue the mutant phenotype in growth, conidiation and penetration (Fig. S7). Similarly, glucose as an energy supplement was also unable to rescue the mutant defects (Fig. S7). Therefore, we concluded the reduced ATP level was not responsible for the phenotypic defects of the mutants.

### ETF mutant *etfb* ^*−*^ shows lipid body accumulation

In human, ETF or ETFDH mutation can lead to lipid storage myopathy because of disruption of fatty acid metabolism^31^. In *M. oryzae,* mutations in the β oxidation proteins ECH1 and MFP1 lead to accumulation of unutilized lipid bodies in the appressorium^32^,^26^. In this study, we investigated whether mutant *etfb*^*−*^ would show similar lipid accumulation via transmission electron microscope (TEM), and found large quantities of dark grey lipid bodies in the conidia and appressoria of mutants, in comparison to Guy11 (Figs 8b and 5b). In parallel, we used the lipophilic probe Bodipy to stain lipid bodies and assessed the lipid accumulation by confocal microscopy. Our results showed extensive lipid bodies as bright green fluorescence in mutant mycelia and conidia, in comparison to Guy11 where the fluorescence signal was much weaker (Fig. 8c).

## Discussion

Electron-transferring flavoprotein ETF and its dehydrogenase ETFDH are highly conserved electron carriers across the fungal and animal kingdoms (Fig. S1). Human ETF and ETFDH mutation leads to deficiency in upstream acyl-CoA dehydrogenases and impacts fatty acids metabolism^19^. Similarly, we discovered ETF and ETFDH deletion in *M. oryzae* also resulted in fatty acids accumulation and utilization defects (Fig. 3a,b). This result suggested the function of ETF and ETFDH in fatty acid metabolism was also conserved between human and *M. oryzae*. Furthermore, the deletion of *M. oryzae* ECH1, which catalyze the downstream of dehydrogenation in mitochondrial fatty acid β oxidation, displayed similar defects in fatty acid metabolism^26^. These results further confirmed the ETF and ETFDH function in fatty acid metabolism.

Human patients carrying ETFDH mutation were effectively cured by taking riboflavin over a long period^33^, while our study did not find the exogenous riboflavin could rescue the defect of *M. oryzae* ETFDH mutant. This probably indicates a different riboflavin-response mechanism between human and fungal ETFDH. However, in *M. oryzae*, exogenously adding NaAC or Na_2_CO_3_ could largely rescue the growth and conidiation defects in ETF and ETFDH mutants (Fig. 3b–d). As weak-base salts, NaAC or Na_2_CO_3_ could counteract acids, so it can be concluded that the growth and conidiation defects of mutants were attributed to the fatty acids accumulation. Moreover, ETF and ETFDH mutants released an unpleasant smell like butyrate when growing on CM medium, while the smell is relatively weak on MM medium. To confirm this smell, butyrate was added to MM medium as sole carbon source to test its effect to fungal growth. As a result, the mutant growth and conidiation were obviously inhibited by butyrate as sole carbon source (Figs 3a and S9a), which is similar to the effect when growing on CM medium. Furthermore, the conidial production of Guy11 was also obviously reduced when growing on butyrate as sole carbon source (Fig. S9b). Therefore, it can be concluded that the impact of excessive fatty acids on mutant conidiation appears to mainly result from the presence of butyrate. These results also suggested growth and conidiation of *M. oryzae* could be impacted by excessive fatty acids.

Although the conidial germination and appressoria formation was normal, ETF and ETFDH mutants failed to cause disease on barley and rice (Fig. 4a). But unlike its effect on improving mutant growth and conidiation, exogenous NaAC could not rescue the mutant pathogenicity defect (Fig. 4a), which implied the fatty acid metabolism disorder was not the only defect in the mutants. As turgor pressure serves as direct force for penetration^7^, the reduced turgor in mutant *etfb*^*−*^ and their different penetration rate on different host surfaces further confirmed the importance of turgor to host penetration (Figs 4b and 5a). Melanin biosynthesis is also essential for appressorium-mediated penetration by keeping high turgor in *M. oryzae*^12^. Here, we discovered melanin biosynthesis was impacted by ETF mutation by displaying a reduced melanin layer in mutant *etfb*^*−*^ (Fig. 5b). This result also corresponded to the white pigmentation of mutant colonies growing on CM culture. Thus we conclude that the low penetration rate of ETF mutant *etfb*^*−*^ on host surface was due to a reduced appressorium turgor which was caused by the thin melanin layer. Consistent with this finding, the *M. oryzae* mutant of ECH1^26^, encoding the Enoyl-CoA hydratase which catalyzes the second step of mitochondrial β oxidation, displayed a similar melanin layer defect.

Despite the low penetration rate, some mutant appressoria still could penetrate into the host cell (Fig. 4b), however, the invasive growth was greatly suppressed (Fig. 4c). Even inoculating wounded host, the mutant *etfb*^*−*^ still could not cause disease (Fig. 6a), which indicated that the penetration defect was not the unique reason for the loss in pathogenicity. ROS produced by host plants could suppress pathogen invasion and intracellular growth^28^,^29^. In mutant-infecting host cells, ROS accumulation was found to be elevated by DAB staining (Fig. 6c). Even without DAB staining, it was also observed that the mutant IH was surrounded by microbodies (Fig. 8d), although the function of these microbodies is unknown. However, by exogenously adding the reducing agent GSH, the unknown microbodies were obviously eliminated and the invasive growth of the mutant was accelerated (Fig. 6d). The penetration rate was not improved by GSH, however (Fig. S7a). Combined with the appressoria turgor defect, we concluded that the pathogenicty defect of the ETF mutant *etfb*^*−*^ mainly resulted from two defects: one is from the low turgor pressure which caused the low penetration rate; another is from not overcoming the host oxidative stress which led to restricted invasive growth.

In summary, we demonstrated that ETF and ETFDH played a pleiotropic role in vegetative growth, conidiation and infection-related development of *M. oryzae* through its role in fatty acids metabolism, turgor generation and host defense response. Additional studies to define the mechanisms for the appressoria melanization defect and the failure to overcome ROS in the mutant should further help to link the metabolic defects to pathogenic development and growth. Our ongoing study of upstream acyl-CoA dehydrogenases will further define the role of the electron-transferring flavoprotein in mediating fungal development and pathogenesis in *M. oryzae*.

## Methods

### Strains and culture conditions

All fungal mutants and transformants described in this study were generated from *Magnaporthe oryzae* Guy11 strain^34^. All culture media were prepared as described previously^9^,^25^. Conidia was harvested from colonies cultured for 12 days on 9 cm Petri plates and counted using a hemacytometer. Appressorium induction was performed by placing conidial suspensions at a concentration of 5 × 10^4 ^ml^−1^ on a hydrophobic surface in a humid environment at 25 °C. Routine bacterial transformations and maintenance of various plasmid vectors were performed in *Escherichia coli* strain DH-5α cultured on LB medium. *Agrobacterium tumefaciens*-mediated transformation (ATMT) and the T-DNA insertion mutant library construction were performed as described previously^35^.

### Vector construction

For constructing gene deletion vectors, 1.5-kb *trpC-hph* cassette with *Kpn*І and *Xba*І ends was cloned to pGEM-T Easy vector (A1360 Promega USA). Then, approximately 1 kb up- and down-stream regions of each gene were amplified from *M. oryzae* genomic DNA and subsequently cloned into the sites flanking the *trpC-hph* cassette. For building gene complementation vectors, the full length of each gene with about 1.5-kb native promoter regions was cloned into pCB1532 vector. The 1.5kb *GFP* sequence with a TrpC-terminator was fused to the C-terminus of the targeted gene for protein localization assays. The mitochondrial marker ATP1-RFP was constructed by fusing RFP to the C-terminus of ATP1 (MGG_07752) driven by the RP27 promoter and loading into pTE11 vector harboring HPH resistance. Peroxisomal targeting protein of *M. oryzae* (MGG_10840)^36^ was fused to RFP in the pTE11 vector to generate peroxysomal markers PTS1-RFP. Primers used for various vector constructing are listed in Supplementary Table S1.

### Fungal transformation

Fungal protoplasts were prepared as described previously^37^. To perform gene deletion transformations, no less than 2 μg deletion vector DNA was introduced into Guy11 protoplasts and transformants were selected for hygromycin resistance. Southern blotting was conducted to confirm the deletion events using the digoxigenin (DIG) high prime DNA labeling and detection starter Kit I (11745832910 Roche Germany). The *HPH* and targeted gene probe were prepared respectively for labeling digested genomes of all fungal strains. Complementation assay was performed by introducing corresponding vector DNA into mutant protoplasts and selecting transformants for chlorimuron-ethyl resistance from pCB1532. Co-localization assays were conducted by co-transformation of ETF-GFP with ATP-RFP and PTS1-RFP into ETF mutant protoplasts.

### Pathogenicity assay

Live rice and barley infection assays were performed as described previously. Conidial suspensions were prepared and sprayed on 15-day-old dwarf Indica rice cv. CO-39 and Golden Promise barley using an airbrush. Post spray-inoculation, plants were placed in a sealed chamber and incubated in a controlled environment with an alternation of 12 h- light and darkness and 90% relative humidity at 25 °C. By 48 hpi, the inoculated plants were moved out and continually incubated for 2–3 days for disease symptom development. Wounded infection was conducted by scratching live barley leaves with a toothpick and inoculated with 20 μl conidial droplets. Infection assays for microscopy were conducted on rice sheaths, barley leaf abaxial epidermis and onion bulb epidermis^38^,^39^.

### Appressorium development assay

Mobilization of Lipid bodies was visualized by staining with Bodipy 493/503 (Invitrogen) as described previously^40^. Conidial suspensions were placed on a hydrophobic surface to induce appressorium development and stained in fresh Bodipy 493/503 solution for 10 min. Lipid body mobilization was observed using a Zeiss Lsm 780 inverted confocal laser scanning microscope. The appressorium turgor was measured using the cytorrhysis assay reported previously^27^. Conidial suspension was prepared and induced for 24 h to allow appressoria maturation. Then water was carefully removed and replaced with an equal volume of glycerol in concentrations of 2.0, 3.0 and 4.0 M. After incubation for 15 min, the number of collapsed appressoria was recorded.

### Transmission Electron Microscope (TEM) Assay

TEM samples were prepared as described^41^. Conidial sample was prepared from 12 day-old colonies and appressorial samples were induced on rice sheaths for 24 h. Then, the tissue and cell samples were prefixed with 0.8% glutaraldehyde and 1% paraformaldehyde in 0.06 M phosphate buffer for 1–2 h on ice, postfixed with 2% (wt/vol) osmium tetroxide for 1 h at room temperature, and dehydrated through an ethanol series. The dehydrated samples were embedded in Spurr resin, processed into ultrathin sections, stained with 2% (wt/vol) uranyl acetate and 0.2 (w/v) lead citrate, and observed under a transmission electron microscope (H-7650, HITACH).

### DAB staining

Host ROS accumulation during infection was detected by staining with DAB as described previously^42^. Conidial suspensions were prepared and inoculated onto barley leaves for 24 h, and then moved to 1 mg/ml DAB solution by incubation at room temperature for 8 h staining. Before observation, samples were destained with washing solution (ethanol/acetic acid = 94/4, v/v) for 2–3 h.

### Total fatty acids and ATP quantitation

In the presence of alkaline copper sulfide, fatty acids generate copper soaps and react with 1,5-diphenylcarbazide producing a cerise color in solution. The solution has a characteristic absorption peak at 550 nm, and the degree of color has a linear relation to the amount of fatty acid. The mycelia sample was prepared and fatty acids extracted and measured by Suzhou Comin Biotechnology Co., Ltd (http://www.cominbio.com/). ATP amount was tested by high performance liquid chromatography (HPLC) according to its absorption peak at 254 nm as described^43^. The mycelia and conidia of strains were prepared and ATP extraction and quantifying services were performed and completed by Suzhou Comin Biotechnology Co., Ltd.

## Additional Information

**How to cite this article**: Li, Y. *et al.* Functional characterization of electron-transferring flavoprotein and its dehydrogenase required for fungal development and plant infection by the rice blast fungus. *Sci. Rep.* **6**, 24911; doi: 10.1038/srep24911 (2016).

## Supplementary Material

Supplementary Information
