# Supplementary material for: Functional characterization of electron-transferring flavoprotein and its dehydrogenase required for fungal development and plant infection by the rice blast fungus
Source: Sci Rep. 2016 Apr 26;6:24911. doi: 10.1038/srep24911 (PMC4845064; doi:10.1038/srep24911)
Supplement: Supplementary Information [file srep24911-s1.pdf]

Functional characterization of electron-transferring flavoprotein and its dehydrogenase required for fungal development and plant infection by the rice blast fungus

Ya Li[1,+], Jindong Zhu[1,+], Jiexiong Hu[1], Xiuli Meng[1], Qi Zhang[1], Kunpeng Zhu[1], Xiaomin Chen[1], Xuehang Chen[1], Guangpu Li[2], Daniel J Ebbole[3], Zonghua Wang[1], Guodong Lu[1,*]

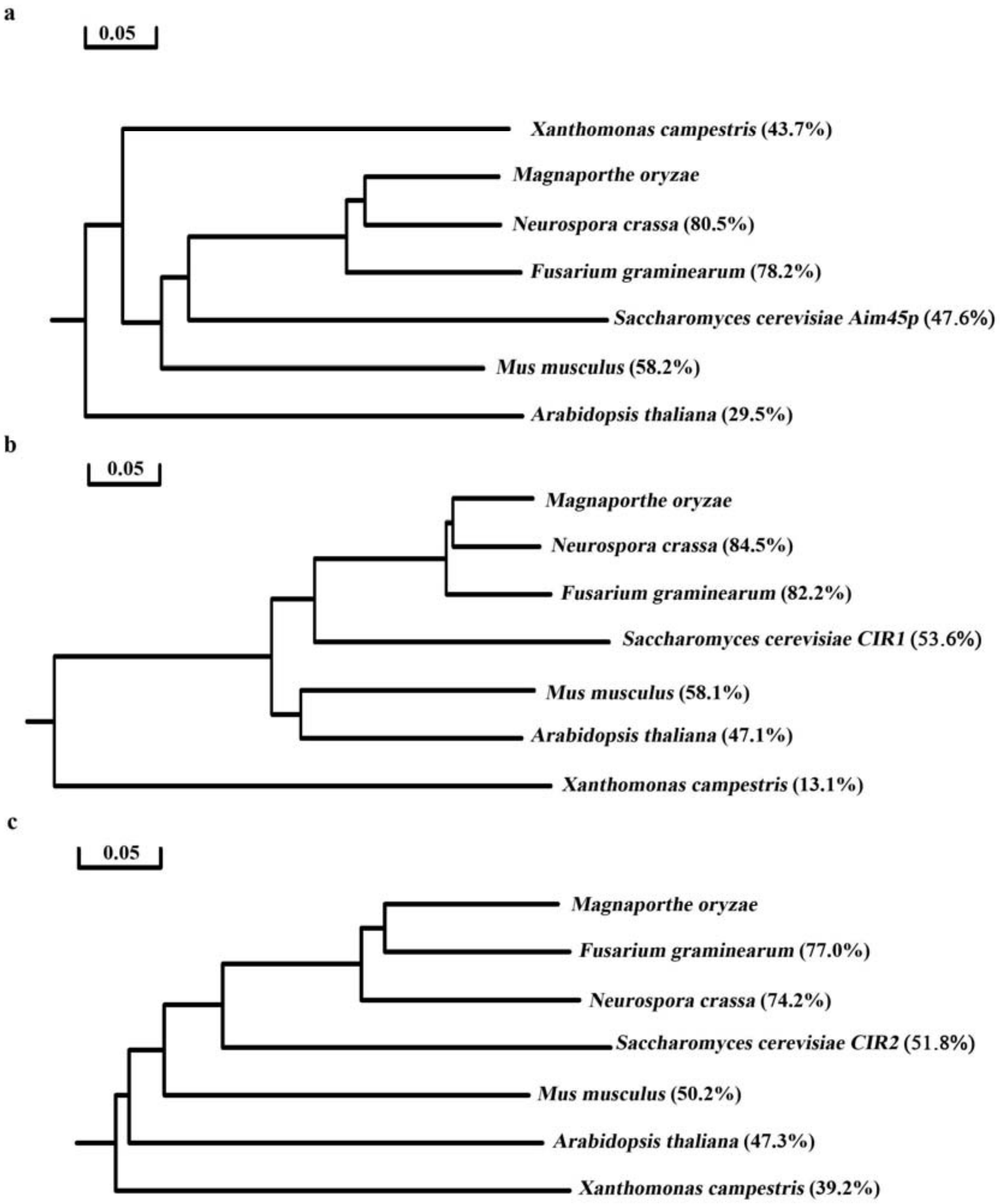

Figure S1. Phylogenetic and homology analysis of ETFA (a), ETFB (b) and ETFDH (c) homologs between different species.

Phylogenetic tree of ETFA, ETFB and ETFDH was constructed by DNAMAN 6.0 version. Four fungi (*Magnaporthe oryzae*, *Neurospora crassa*, *Fusarium graminearem* and *Saccharomyces cerevisiae*), one bacterium (*Xanthomonas campestris*), one plant (*Arabidopsis thaliana*) and one animal (*Mus musculus*) were selected to analyze the evolution and homology of ETFA, ETFB and ETFDH. The percentage in brackets indicated the amino acid similarity between *M. oryzae* ETFA, ETFB and ETFDH and their homologs.

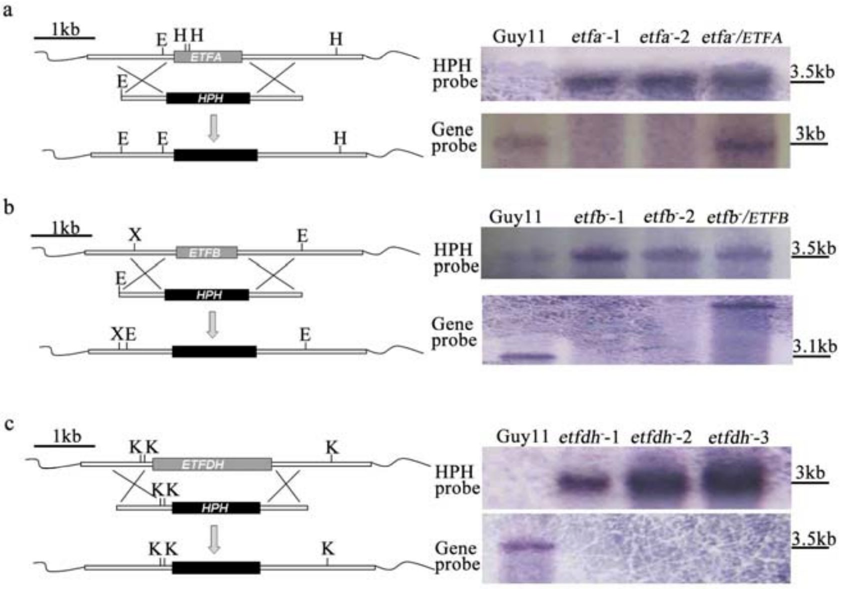

Figure S2. Southern blot confirming the deletion of ETF and ETFDH encoding genes.

About 1-kb of *HPH* DNA and target gene ORF region was made as molecular probes for blotting. In *ETFA* assay of **a**, the genomes were digested by *Eco*RⅠ and *Hin*dⅢ and blotted with *HPH* probe, the mutants displayed one brand of ~3.5-kb, while Guy11 had no brand; and then the genomes were digested by *Hin*dⅢ and blotted with gene probe, the mutants had no brand, while Guy11 displayed a brand of ~3.0-kb. In *ETFB* assay of **b**, the genomes were digested by *Eco*RⅠ and *Xba*Ⅰ. When blotted with *HPH* probe, the mutants displayed one brand of ~3.5-kb, while Guy11 had no brand; when blotted with gene probe, the mutants had no brand, while Guy11 displayed a brand of 3.1-kb. In *ETFDH* assay of **c**, the genomes were digested by *Kpn*Ⅰ. When blotted with *HPH* probe, the mutants displayed one brand of ~3.0-kb, while Guy11 had no brand; when blotted with gene probe, the mutants had no brand, while Guy11 displayed a brand of 3.5-kb.

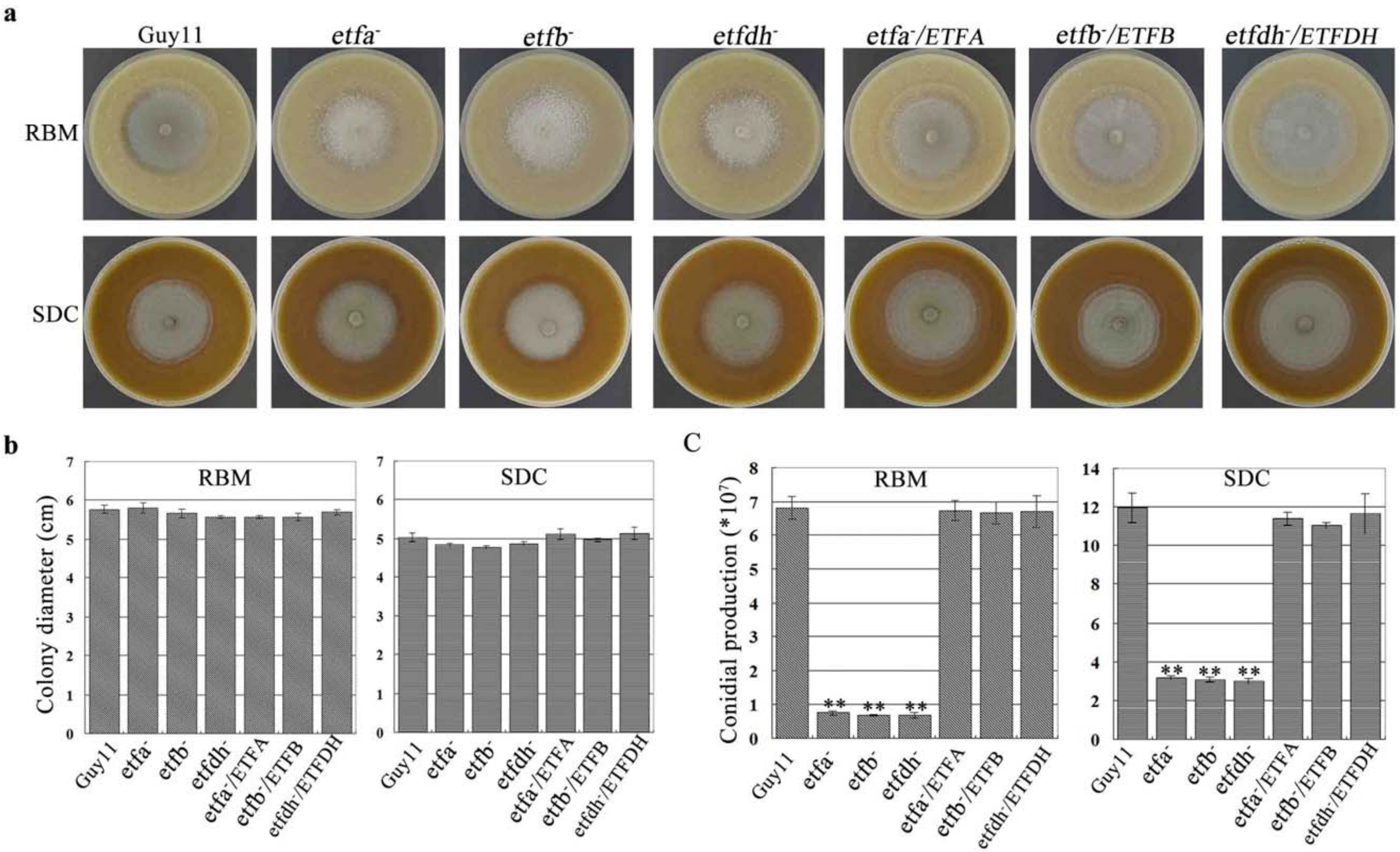

a
Guy11
etfa-
etfb-
etfdh-
etfa-/ETFA
etfb-/ETFB
etfdh-/ETFDH
RBM
SDC
b
Colony diameter (cm)
RBM
SDC
Guy11
etfa-
etfb-
etfdh-
etfa-/ETFA
etfb-/ETFB
etfdh-/ETFDH
C
Conidial production (*10⁷)
RBM
SDC
**

Figure S3. The growth and conidiation defects of ETF and ETFDH mutants by growing on RBM and SDC medium.

a. Colony morphology of ETF and ETFDH mutants growing on RBM and SDC medium for 10 days.

b. Bar chart showing the growth rate of ETF and ETFDH mutants growing on RBM and SDC medium for 10 days. No obvious growth change was observed in mutant growing on RBM and SDC medium. Mean and deviation were calculated from three independent replicates.

c. Bar chart showing the conidial production of ETF and ETFDH mutants growing on RBM and SDC medium for 12 days in one 9cm plate. The conidial production of mutants reduced to ~10% of Guy11 on RBM and SDC medium. Mean and deviation were calculated from three independent replicates. Significant differences are indicated by stars (**, $P < 0.01$; t test).

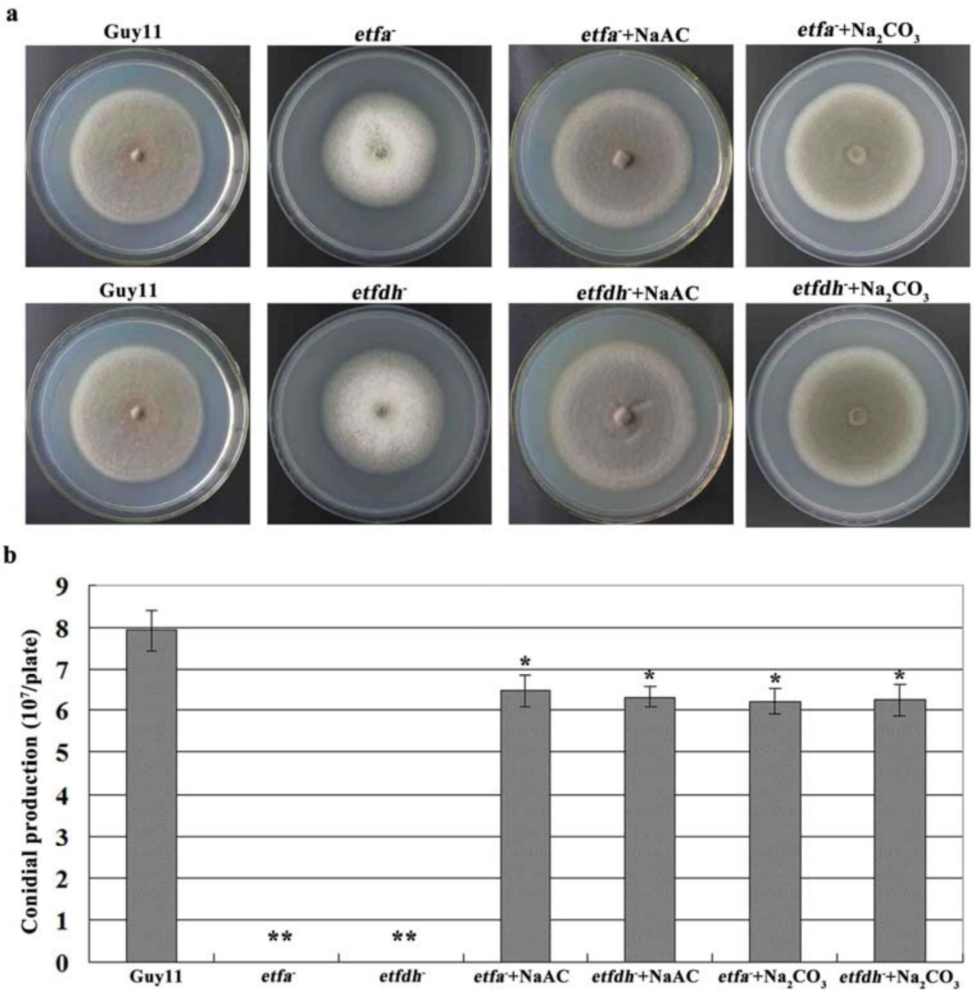

Figure S4. The effect of NaAc and Na_2Co_3 to growth and conidiation of mutant *etfa*$^-$ and *etfdh*$^-$.

a. The effect of NaAc to growth of mutant *etfa*$^-$ and *etfdh*$^-$. The mutant colony color and size were almost completely recovered.

b. The effect of NaAc and Na_2Co_3 to conidiation of mutant *etfa*$^-$ and *etfdh*$^-$. The mutant conidial production was largely restored. Mean and deviation were calculated from three independent replicates. Significant differences are indicated by stars (**, P < 0.01; t test).

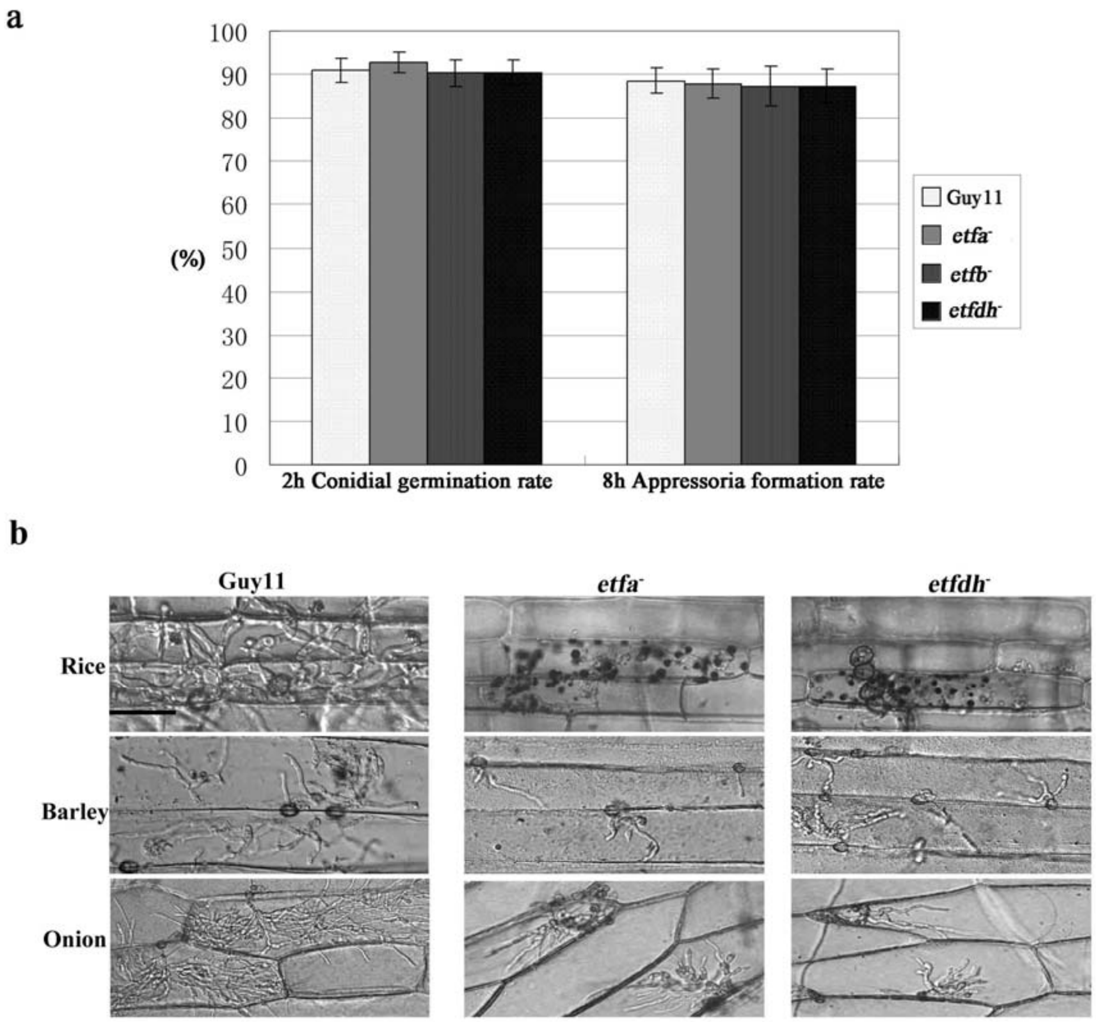

Figure S5. The conidial germination, appressoria formation and invasive growth of ETF and ETFDH mutants.

a. The conidial germination and appressoria formation was normal in ETF and ETFDH mutants in comparison to Guy11.

b. The invasive growth of mutant *etfa*$^{-}$ and *etfdh*$^{-}$ was restricted in rice, barley and onion cells.

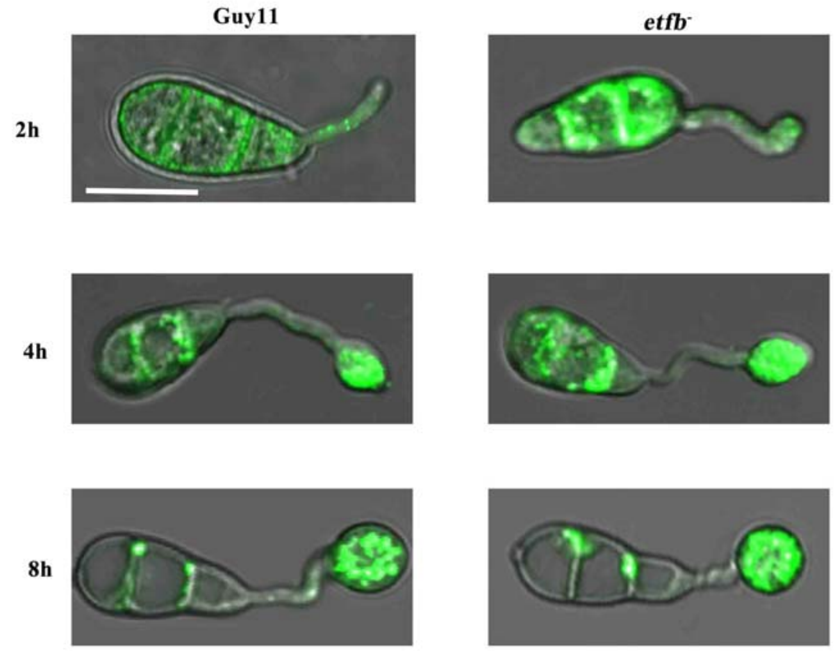

Figure S6. Lipid body mobilization detected by Bodipy staining during appressoria development.

The appressoria were induced on hydrophobic surface as time intervals and stained by Bodipy for 15 minutes before observation by confocal microscopy. From 2h to 8h development, the lipid bodies were normally transported to appressorium from conidia whether in mutant *etfb*$^-$ or Guy11 strain. More intensive lipid bodies which emit brighter fluoresces were present in mutant *etfb*$^-$. Bar, 10μm.

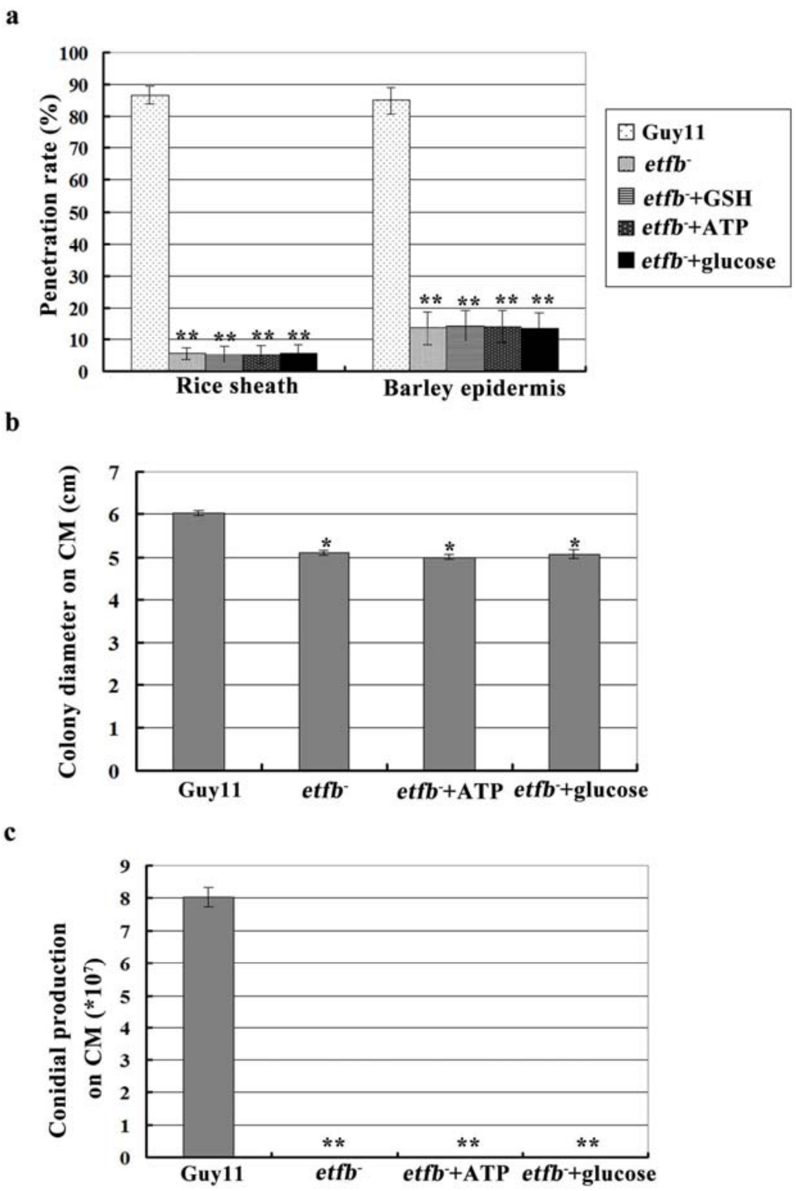

Figure S7. The effect of exogenous chemicals to ETF and ETFDH mutants

a. Bar chart showing the effect of exogenous GSH (5 mM), ATP (5 mM) and glucose (2.5%) to mutant penetration. Exogenously adding these chemicals to *etfb*$^-$ could not improve the penetration defect on rice sheath and barley epidermis. Mean and deviation were calculated from three independent replicates. Over 100 appressoria were counted for calculating the penetration rate in one replicate. Significant differences are indicated by stars (**, $P < 0.01$; t test).

b. Bar chart showing the effect of exogenous ATP and glucose to mutant growth defect on CM. The colony size of *etfb*$^-$ was not recovered by adding ATP and glucose. Mean and deviation were calculated from three independent replicates. Significant differences are indicated by stars (*, $P < 0.05$; t test).

c. Bar chart showing the effect of exogenous ATP and glucose to mutant conidial production on CM. The conidial production of *etfb*$^-$ was not recovered by adding ATP and glucose. Mean and deviation were calculated from three independent replicates. Significant differences are indicated by stars (**, $P < 0.01$; t test).

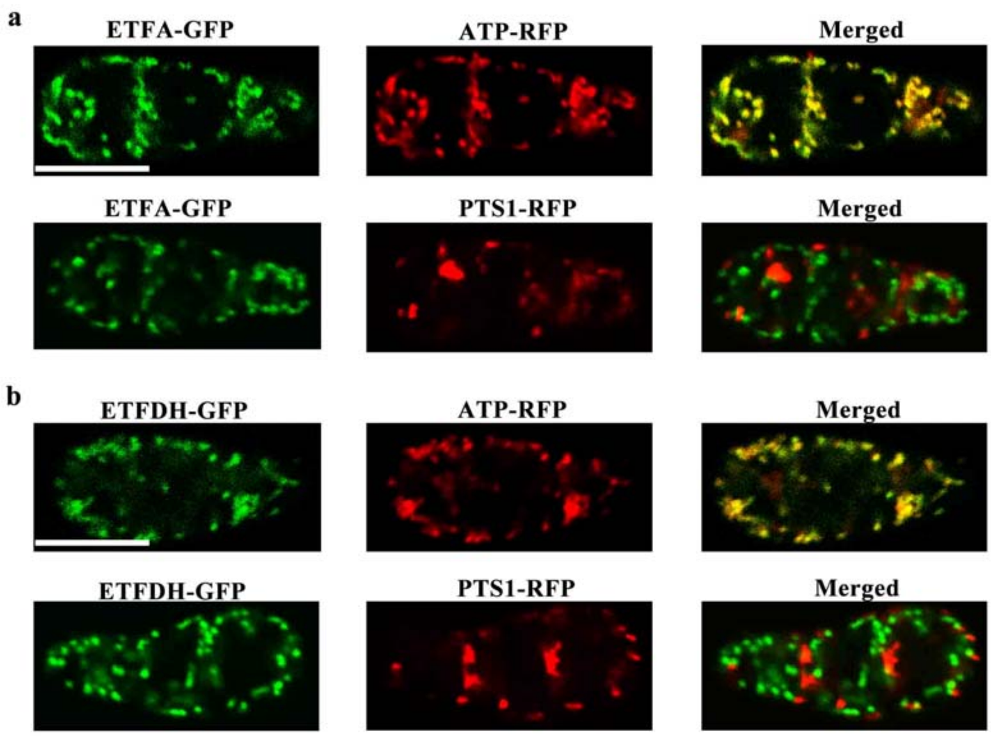

Figure S8. The ETFA and ETFDH localization determined by co-localization analysis.
The co-localization of ETFA-GFP (a) and ETFDH-GFP (b) to mitochondrial marker ATP-RFP and peroxisomal marker PTS1-RFP observed by confocal microscopy. ETFA-GFP and ETFDH-GFP almost completely co-localize with ATP-RFP, not PTS1-RFP, indicating a mitochondrial localization of ETFA and ETFDH. Bar, 10μm.

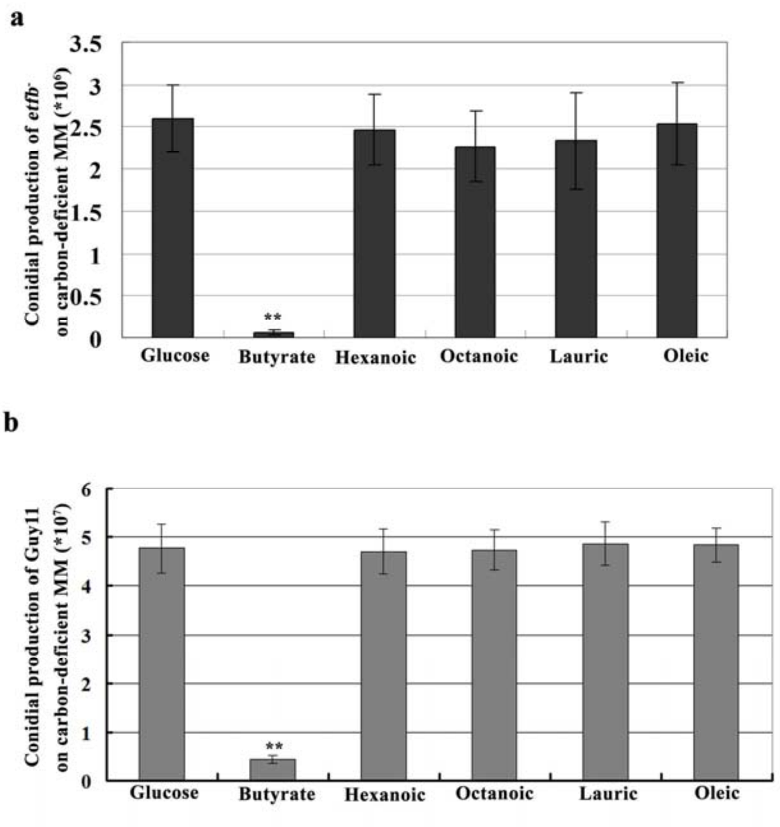

Figure S9. The conidial production of mutant *etfb*$^-$ (a) and Guy11 (b) by growing on carbon-deficient MM with fatty acids as sole carbon source.

Glucose (50 mM), Butyrate (10 mM), Hexanoic (2.5 mM), Octanoic (1 mM), Lauric (2.5 mM), oleic (2.5 mM). Mean and deviation were calculated from three independent replicates. Significant differences are indicated by stars (**, P < 0.01; t test).

Table S1. Primers used in this study

Name	Sequence	Usage
MG1719-Up-EcoR1-F	GGAATTCCATTCTCCAGAGTCTCCTTG	*ETFA* deletion
MG1719-Up-Xba1-R	GCTCTAGACTGTGCTGTGAATGCGTTGC	*ETFA* deletion
MG1719-Dw-Kpn1-F	GGGGTACCAACAGAGTAGAGTACGGACC	*ETFA* deletion
MG1719-Dw-Pst1-R	AACTGCAGCATGCAGGCTGCCTTTACCAT	*ETFA* deletion
MG1719-Probe-F	TGGTGGTCGAGAACAGCGCATAC	*ETFA* probe
MG1719-Probe -R	TTGCGCCCGAAATACCAACAGCC	*ETFA* probe
MG1719-Promoter-Xba1-F	GCTCTAGAAATGTGGTGGTTCCATCCAT	*ETFA* complementation
MG1719-ORF-Spe1-R	GACTAGTTGACTTGCCGAGCTTCTCGGT	*ETFA* complementation
MG1719-ORF-Spe1-R2	GACTAGTCTTGCCGAGCTTCTCGGT	ETFA localization
MG1744-Up-EcoR1-F	GGAATTCGGTATGTGGAATCTACGTCT	*ETFB* deletion
MG1744-Up-BamH1-R	CGGGATCCCGAATGATGATGATGTCGTG	*ETFB* deletion
MG1744-Dw-Kpn1-F	GGGGTACCATACACACCAAGACACCACT	*ETFB* deletion
MG1744-Dw-Pst1-R	AACTGCAGCTATCCCAGTTTCTTGCGCT	*ETFB* deletion
MG1744-Probe-F	ATGAACCCATTCGATGAGCTCTC	*ETFB* probe
MG1744-Probe-R	TCAGTCCATCGACATCCTCAACC	*ETFB* probe
MG1744-Promoter-Xba1-F	GCTCTAGAGCTTTGGGGAAGATGTTGG	*ETFB* complementation
MG1744-ORF-EcoR1-R	GGAATTCTTACAATGCCCCCAGTTCCT	*ETFB* complementation
MG1744-ORF-Pst1-R	AACTGCAGCAATGCCCCCAGTTCCTTGA	ETFB localization
MG8880-Up-BamH1-F	CGGGATCCGTCCGTGAGTAACCAGGCAAGT	*ETFDH* deletion
MG8880-Up-BamH1-R	CGGGATCCAGGAGGGGGCAAGGGTCACTCT	*ETFDH* deletion
MG8880-Dw-Kpn1-F	GGGGTACCGTCACTGAAACCACCCCCTATGT	*ETFDH* deletion
MG8880-Dw-Pst1-R	AACTGCAGCAGGTCTTCAAGCAGCTCTCCCT	*ETFDH* deletion
MG8880-Probe -F	GAGCGATGAGGTGGATGTTT	*ETFDH* probe
MG8880-Probe-R	ACTGGCCCAAACTAACGATG	*ETFDH* probe
MG8880-Promoter-F	GATAGACTACCCCGAGATATCATGC	*ETFDH* complementation
MG8880-ORF-Not1-R	TTGCGGCCGCTTACTTTTGAGCGTTGATTTGG	*ETFDH* complementation
MG8880-ORF-EcoR1-R	CGGAATTCCTTTTGAGCGTTGATTTGGAAC	ETFDH localization
MG7752-Promoter-EcoR1-F	GGAATTCCAAGAGCAGAGTCGGTACCA	ATP localization
MG7752-ORF-Spe1-R	GGACTAGTGGCGATGAAGCTCTTGGTGA	ATP localization
MG10840-Promoter-SpeI-F	GGACTAGT CAGTACGGCAATGTTGTCGAC	PTS1 localization
MG10840-ORF-SpeI-R	GGACTAGT TGGTTGTGATAACGGCAGACG	PTS1 localization
RFP-SpeI-F	GGACTAGT TGGTGAGCGTGTCTCCCGCC	ATP and PTS1 fusion
RFP-NotI-R	GCGGCCGCGGCGATGAAGCTCTTGGTGA	ATP and PTS1 fusion
GFP-EcoR1-F	GGAATTCATGGTGAGCAAGGGCGAGGA	ETF and ETFDH fusion
GFP-Kpn1-R	GGGGTACCTTACTTGTACAGCTCGTCCA	ETF and ETFDH fusion
